# Corrosion inhibition properties of schiff base derivative against mild steel in HCl environment complemented with DFT investigations

**DOI:** 10.1038/s41598-023-36064-w

**Published:** 2023-06-02

**Authors:** Nadia Betti, Ahmed A. Al-Amiery, Waleed Khalid Al-Azzawi, Wan Nor Roslam Wan Isahak

**Affiliations:** 1 Materials Engineering Department, University of Technology-Iraq, P.O. Box: 10001, Baghdad, Iraq; 2 Department of Chemical and Process Engineering, Faculty of Engineering and Built Environment, Universiti Kebangsaan Malaysia (UKM), 43000 Bangi, Selangor Malaysia; 3 Energy and Renewable Energies Technology Center, University of Technology-Iraq, Baghdad, 10001 Iraq; 4 Al-Farahidi University, Baghdad, 10001 Iraq

**Keywords:** Chemical engineering, Materials chemistry, Theoretical chemistry

## Abstract

There is growing interest in using corrosion inhibitors and protective treatments to limit the degradation of mild steel, leading to the development of numerous Schiff bases as cutting-edge inhibitors. In this study, the effectiveness of a Schiff base, 3-((5-mercapto-1,3,4-thiadiazol-2-yl)imino)indolin-2-one (MTIO), to prevent mild steel corrosion in HCl was investigated using weight loss measurements, potentiodynamic polarization measurements, electrochemical impedance spectroscopy techniques, and surface characterization. The experimental results showed that 0.5 mM MTIO exhibited a satisfactory inhibitor efficiency of 96.9% at 303 K. The MTIO molecules physically and chemically adsorbed onto the mild steel surface following the Langmuir model, forming a compact protective film attributed to the presence of a thiazole ring in the MTIO structure. Theoretical calculations were combined with experimental techniques to investigate the anticorrosion performance and mechanism of inhibition.

## Introduction

Mild steel is commonly used to make structural components^1^, but it is particularly prone to environmental corrosion^2^, leading to significant economic losses^3^. Therefore, ongoing research aims to develop corrosion inhibitors^4,5^ for industrial applications, especially in the oil and gas industries^6–9^. An efficient inhibitor requires a heterocyclic ring and/or heteroatoms such as nitrogen, oxygen, sulfur, and pi-systems to coordinate with the d-orbital of iron and form coordination bonds^10–12^. Organic inhibitors are environmentally safe and exhibit good anti-corrosion characteristics^13–15^. The aromatic thiadiazole, containing heteroatoms of sulphur and nitrogen, along with isatin, which contains oxygen and nitrogen, serve as electron donors. Previous research reported that 0.01 M of 2-amino-5-mercapto-1,3,4-thiadiazole achieved 99% inhibition efficiency for mild steel corrosion in 1 M HCl^16^. Al-Amiery et al. investigated the corrosion protection of a novel Schiff base, 5,5'-((1Z,1'Z)-(1,4-phenylenebis(methanylylidene))bis(azanylylidene))bis(1,3,4-thiadiazole-2-thiol) (PBB), containing an imine bond and a phenyl ring, and achieved 95.16% inhibition efficiency for mild steel in 1 M HCl solution^17^. Comparing the two studies reveals that the chemical structures of the inhibitors used in both studies contain thiadiazole, but the addition of an imine bond and a phenyl ring in PBB resulted in slightly lower inhibition efficiency than that achieved with 2-amino-5-mercapto-1,3,4-thiadiazole. Anticorrosion behavior has been evaluated, but it is still unclear which substituents contribute most to corrosion inhibition. Experimental research is costly and time-consuming, so theoretical approaches, currently supported by sufficient software and technology, have been adopted to overcome such issues. A particle's capability to prevent corrosion is dependent on its charge distribution, which can be precisely determined through theoretical research, as the adsorption site during corrosion inhibition can be predicted through the application of quantum chemical simulations^18^. Issues regarding analytical outcomes relating to the interactions of natural compounds with metallic surfaces can be answered using quantum chemistry calculations^19^. Density functional theory (DFT) can be used to provide a complete description of inhibitor behavior concerning its orientation and structure, as well as how the inhibitor adsorbs to the metal surface^20^. For example, Hadisaputra et al. used DFT to forecast the effectiveness of coumarins and caffeine as metallic anticorrosion compounds^21^. The degree to which organic corrosion inhibitors interact with metallic surfaces depends on the donor- and electron-withdrawing sites, as well as the position^22^.

There is much research on mild steel anticorrosion compounds in corrosive environments^13^, such as organic compounds with structural moieties including tetrazoles, imidazoles, triazoles, quinolones, pyridines, Schiff bases, quaternary ammonium salts, and Mannich bases^23–30^. A particularly significant class of corrosion inhibitors utilized in the highly concentrated acidic environment is Schiff bases made from thiadiazoles and aldehydes^31,32^. The production of a protective barrier on a steel surface is significantly influenced by the Schiff base produced from thiadiazole molecules. Although numerous studies of corrosion inhibitors for mild steel have been published, most are performed in strong acid environments, such as hydrochloric acid, sulfuric acid, and phosphoric acid^33,34^. By establishing a protective coating, becoming adsorbed, or producing an insoluble compound on the steel surface, the inhibitor blocks the active site to prevent corrosion^35^, so the inhibitor efficiency is influenced by the inhibitor structure. The adsorption of the inhibitor to the steel surface is determined by the presence of phosphorus, sulfur, oxygen, and nitrogen atoms, as well as pi-electrons (double bonds)^36^. Simplified correlations between molecular electronic parameters and corrosion inhibition efficiencies are often evaluated. In many studies, a relationship has been found between these electronic parameters and the corrosion inhibition efficiencies of an organic corrosion inhibitor. But some researchers have a different opinion depending on their research results, where 12 various molecular electronic parameters were examined, and no one of the tested parameters showed any significant association with protection performance. The observed correlations between these characteristics and inhibitory performance, established for only a few compounds and widely published in the publications, are thus called into question by their findings^37^.

**Figure 1:**
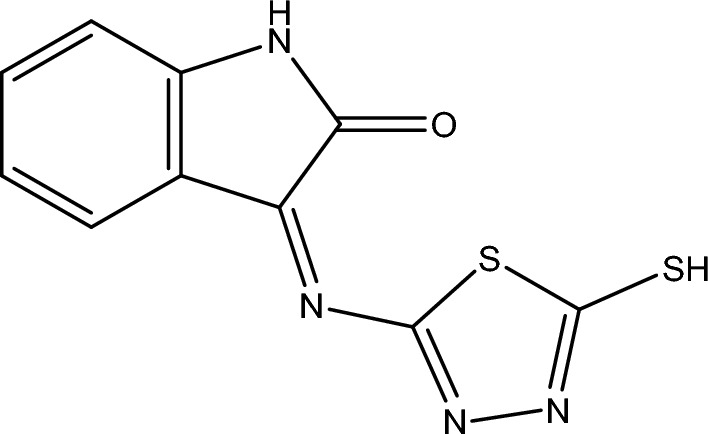
The chemical structure of MTIO.

Corrosion of metallic substrates is a major challenge faced in various industrial processes. Schiff bases have emerged as promising candidates for corrosion inhibitors due to their excellent adsorption and film-forming properties. In particular, the Schiff base 3-((5-mercapto-1,3,4-thiadiazol-2-yl)imino)indolin-2-one (MTIO) has shown great potential as a corrosion inhibitor for metallic substrates in acidic media. Although several other Schiff bases have been reported in the literature, the corrosion inhibition efficiency of MTIO has not been extensively studied using both experimental and theoretical approaches. Therefore, in this study, we investigated the corrosion inhibition performance of MTIO using weight loss measurements, potentiodynamic polarization (PDP) measurements, electrochemical impedance spectroscopy (EIS) techniques, and surface characterization. Additionally, we used density functional theory (DFT) calculations to gain insights into the molecular mechanism of corrosion inhibition. The results of this study provide new insights into the effectiveness of MTIO as a corrosion inhibitor and contribute to the development of more effective corrosion prevention strategies for metallic substrates in acidic environments. Herein, we report the investigation of the corrosion prevention properties of MTIO, a Schiff base-type anticorrosion agent depicted in Fig. 1. The effectiveness of MTIO in preventing corrosion of metallic substrate steel in corrosive media was studied using DFT.

## Materials and methods

### Metallic sample preparation

An X-Ray fluorescence (XRF) spectrometer was employed to analyse the chemical composition of the mild steel coupons with the following weight ratios: 0.21 C, 0.05 Mn, 0.09 F, 0.05 S, 0.01 Al, 0.38 Si, and balance Fe. Specimens with a cubic area of ${1 cm}^{2}$ were used for PDP and EIS measurements, while specimens of $2.50 x 2.00 x 0.03 cm$ were employed in weight loss measurements. The samples were prepared according to ASTM G1-03^38^, and a series of silicon carbide plates (Grade 320–1200) was used to scrape the surfaces. The specimens were rinsed in double-distilled water followed by acetone and oven-dried.

### Test media preparation

The corrosive environment was HCl 1 M, prepared by the dissolution of 37% hydrochloric acid in double-distilled water. Various concentrations of the inhibitor (0.1, 0.2, 0.3, 0.4, 0.5, and 1.0 mM) were prepared by suitable dilution in 1 M HCl.

### Weight loss techniques

The weight loss measurements were conducted based on NACE TM0169/G31^39^. The manufactured metallic substrate coupons were weighed and placed in 500 mL beakers with 400 mL of 1 M HCl with the addition of different concentrations of tested inhibitor ($0.1, 0.2, 0.3, 0.4, 0.5,\mathrm{ and }1\mathrm{ mM}$) at 303 K in a water bath for 1, 5, 10, 24, and 48 h. Then, the corrosion products were cleaned off the surface of the specimens before dried a period after. The samples were weighted to determine the weight loss ($\mathrm{W}$). The metallic substrates were also immersed in inhibited corrosive media for 5 h with the different inhibitor concentrations at $303, 313, 323\mathrm{ and }333\mathrm{ K}$ utilizing a water bath to determine the influence of temperature. To ensure the accuracy of the results, each test was performed three times, and the average was recorded. Rate of corrosion rate (${\mathrm{C}}_{\mathrm{R}})$ was calculated as follows in Eq. (1):

1 $${C}_{R}=\frac{87.6W(mg)}{tad}$$

where a is the coupon area (${cm}^{2}$), d is the coupon density (${g.cm}^{-3}$), and t is the immersion period (h).

The inhibition efficiency was determined according to Eq. (2):

2 $$IE\%=\frac{{C}_{Ro}-{C}_{Ri}}{{C}_{Ro}}\times 100$$

where ${C}_{Ro}$ is the corrosion rate in uninhibited solution, ${C}_{Ri}$ is the corrosion rate in inhibited solution.

Based on Eq. (3), the θ (surface coverage area) was determined for different inhibitor concentrations in corrosive solution from the mass lost measurements:

3 $$\theta =\frac{{C}_{Ro}-{C}_{Ri}}{{C}_{Ro}}$$

### EIS and PDP techniques

The Gamry Instrument ($reference\,600\,potentiostat/galvanostat/ZRA-\,model (Gamry,\,Warminster,\,PA,\,USA)$) was used to conduct the electrochemical techniques according to ASTM G1-03^38^. The metallic substrate samples were used as working electrodes, and the electrochemical measurements were initiated 30 min after the working electrode was exposed to an acidic medium, while maintaining a steady-state potential of 303 K. To use the Gamry Echem Analyst tool, the major influence potential was altered from $0.25\,to +0.25\,V\,SCE$ at a scanning rate of ${0.5 mV s }^{-1}$. All impedance values were then fitted to the relevant equivalent circuits (ECs). The working electrode, counter electrode, and reference electrode (SCE) were the main electrodes that composed the Gamry water-jacketed glass cell. A saturated calomel electrode was used as the reference electrode^40^.

### Adsorption isotherm

Additional information on the properties of the substances under investigation can be found throughout the adsorption isotherms. Several adsorption isotherms, including Langmuir (Eq. 4), Temkin (Eq. 5), and Frumkin (Eq. 6), should be utilized to estimate the inhibitor's degree of surface covering (θ) in order to choose the best isotherm model. Thus, the parameter (θ) for various inhibitor doses in 1 M HCl solution were examined using weight loss techniques.

4 $$\frac{C}{\theta }=\frac{1}{{K}_{ads}}+C$$

5 $$log\frac{\theta }{C}=logk+\alpha \theta$$

6 $$log\frac{\theta }{1-\theta }=2.303logk+2\alpha \theta$$

where K_ads_ is the adsorption–desorption constant and θ is the surface coverage.

### SEM

The metallic substrate coupons were immersed in the uninhibited and inhibited solutions for 5 h and then observed using a Compact FESEM (Zeiss MERLIN) at a resolution of 0.8 nm (15 kV/1.6) in STEM mode at the Electron Microscopy Unit of Universiti Kebangsaan Malaysia in Selangor. After exposure to the acidic media with and without the inhibitor, the coupons were washed with distilled water, dried, and analyzed by SEM.

### Quantum chemical calculations

Gaussian 09 was used to run the quantum mechanical calculations^20^. The B3LYP functional was used to optimise the organic molecule structure in the gaseous state, with the justification set to “6-31G +  + ” (d,p). Relations (7) and (8) were used to compute the ionisation potential (I) and electron affinity (A), which are related to ${E}_{HOMO}$ and ${E}_{LUMO}$, correspondingly, according to Koopman's theorem^41^.

7 $$I=-{E}_{HOMO}$$

8 $$A=-{E}_{LUMO}$$

In order to determine the electronegativity (χ), softness (σ) and hardness (η), Eqs. (9) to (11) were utilized.

9 $$\chi =\frac{I+A}{2}$$

10 $$\eta =\frac{I-A}{2}$$

11 $$\sigma ={\eta }^{-}$$

Equation (12) was applied to calculate the fractional number of transported electrons ($\Delta N$),^42^:

12 $$\Delta N=\frac{{\chi }_{Fe}-{\chi }_{inh}}{2\left({\eta }_{Fe}+{\eta }_{inh}\right)}$$

The electronegativity value for iron (χ_Fe_) was equal to 7 eV, whereas the hardness of iron η_Fe_ was equal to 0 eV as in Eq. (13):

13 $$\Delta N=\frac{7-{\chi }_{inh}}{2\left({\eta }_{inh}\right)}$$

## Results and discussion

### Weight loss technique

#### Concentration effects

**Figure 2:**
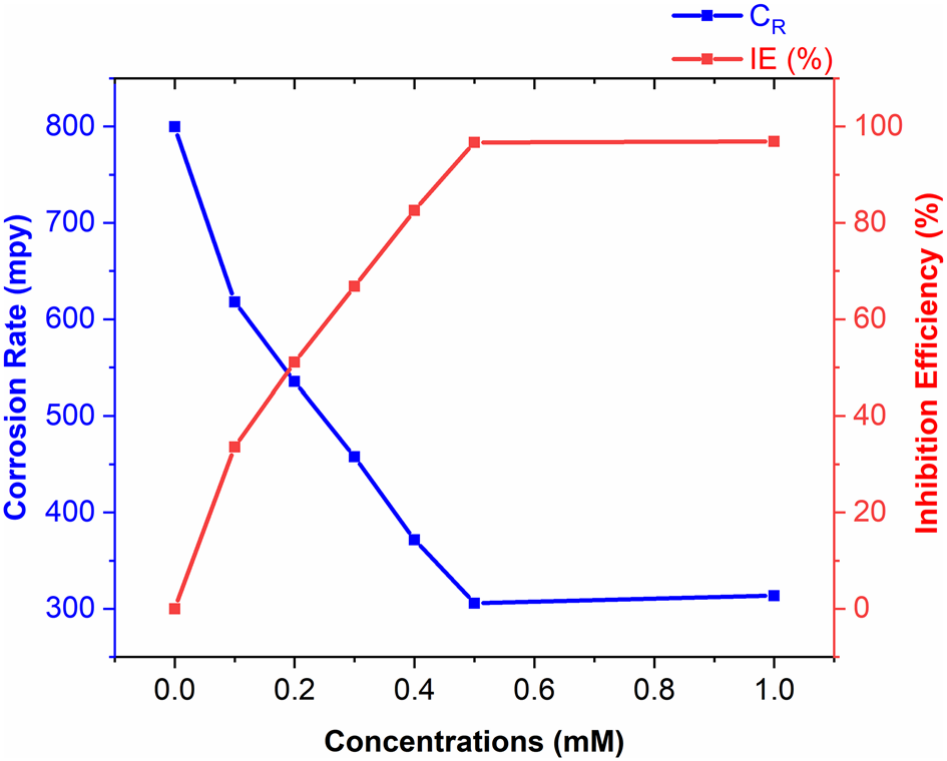
The effect of inhibitor concentration on the ${C}_{R}$ and $IE\%$ of metallic substrate subjected to 1 M HCl for 5 h at 303 K.

${C}_{R}$ And IE% are presented in Fig. 2, showing that the inhibitor's ability to prevent corrosion increased with increasing concentration up to 0.5 mM. Beyond this concentration, the inhibition efficiency remained constant, indicating that the surface coverage achieved equilibrium and further concentration increase would not provide any additional gain in terms of efficiency.

#### Immersion time effect

**Figure 3:**
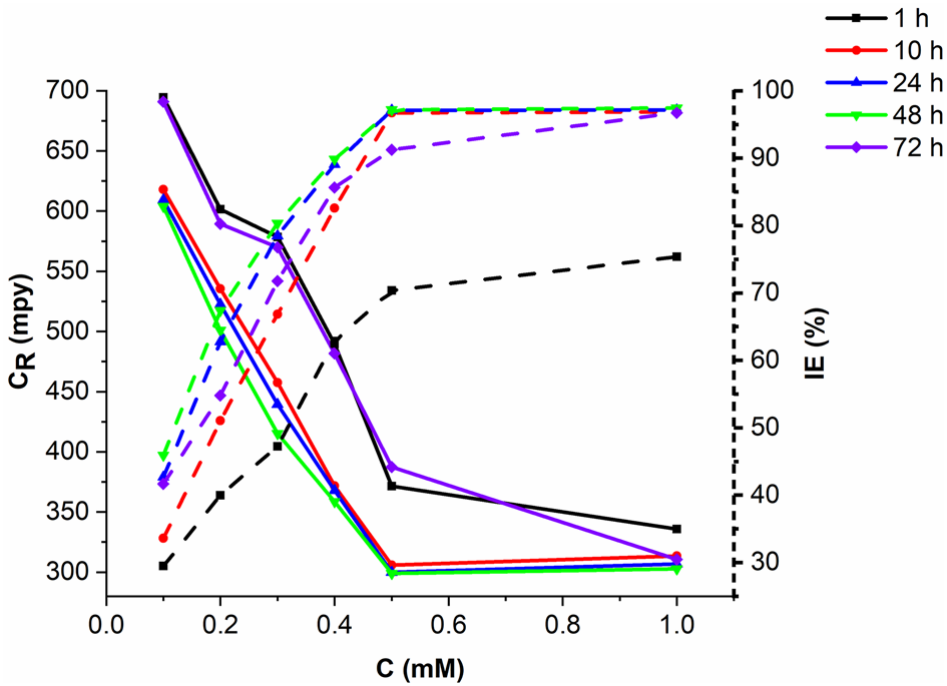
The effect of immersion time and various inhibitor concentrations on the C_R_ and IE% of mild steel in 1 M HCL at 303 K.

The effect of exposure period on the IE was investigated by immersing the mild steel specimens in 1 M HCl with/without various inhibitor concentrations for $1, 5, 10, 24 and 48 h at 303 K$. As shown in Fig. 3, there was a sharp increase in the IE up to 5 h followed by a steady increase up to 24 h, then the IE begins to decline becoming stable after 48 h. The IE increases as the number of inhibitor molecules adhering to the metallic substrate surface increases to form a protective barrier. The action of van der Waals forces between inhibitor molecules will cause the desorption of some inhibitor molecules, resulting in a reduction in the active area and, consequently, reduced inhibitory efficacy. The stability of the adsorbed protective layer in the presence of 1 M HCl is demonstrated by the relatively high IE demonstrated during prolonged exposure^43^.

#### Temperature effects

**Figure 4:**
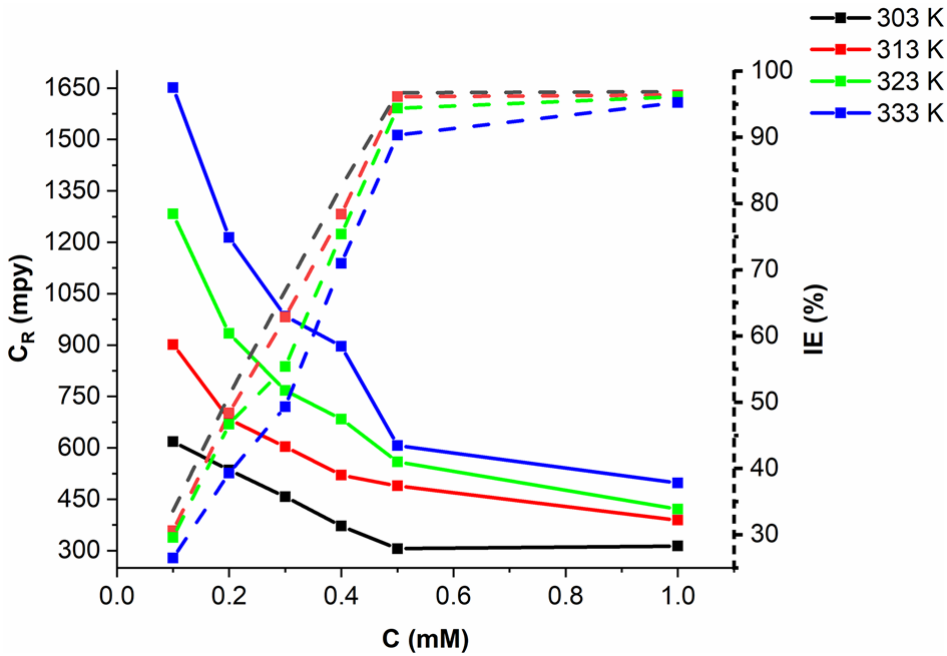
Temperature effect in 1 M HCl in various MTIO concentrations after 5 h immersion.

Weight loss tests were conducted at different temperatures (303, 313, 323, and 333 K) to evaluate the effect of temperature on the inhibitory performance of MTIO, as illustrated in Fig. 4. The results show that the corrosion rate (${C}_{R}$) of the metallic substrate in 1 M HCl increases with temperature, even in the presence of the tested inhibitor at the same concentration. This is because as the temperature increases, the HCl molecules become more energetic and agitated, leading to an increase in conductivity and, consequently, a higher corrosion rate. Despite the increasing corrosion rate with temperature, the addition of MTIO still provides some degree of corrosion protection, albeit to a lesser extent compared to lower temperatures^44^.

As increasing temperature, the IE decreases due to the increase in the ${C}_{R}$ and corrosion occurs due to the removal of the inhibitor particles from the metal surface, thereby increasing the surface of metallic substrate in contact with the acid environment and hence the ${C}_{R}$. The relation of Arrhenius^45^ (Eq. 14) is a significant example of the activation energy ${(E}_{a} )$ apparent of the ${C}_{R}$ and process of corrosion:

14 $${C}_{R}=A\mathrm{exp}\frac{{-E}_{a}}{RT}$$

where R is the gas constant ($8.314\,J\,{mol}^{-1} {K}^{-1}$), and A is the Arrhenius parameter.

**Figure 5:**
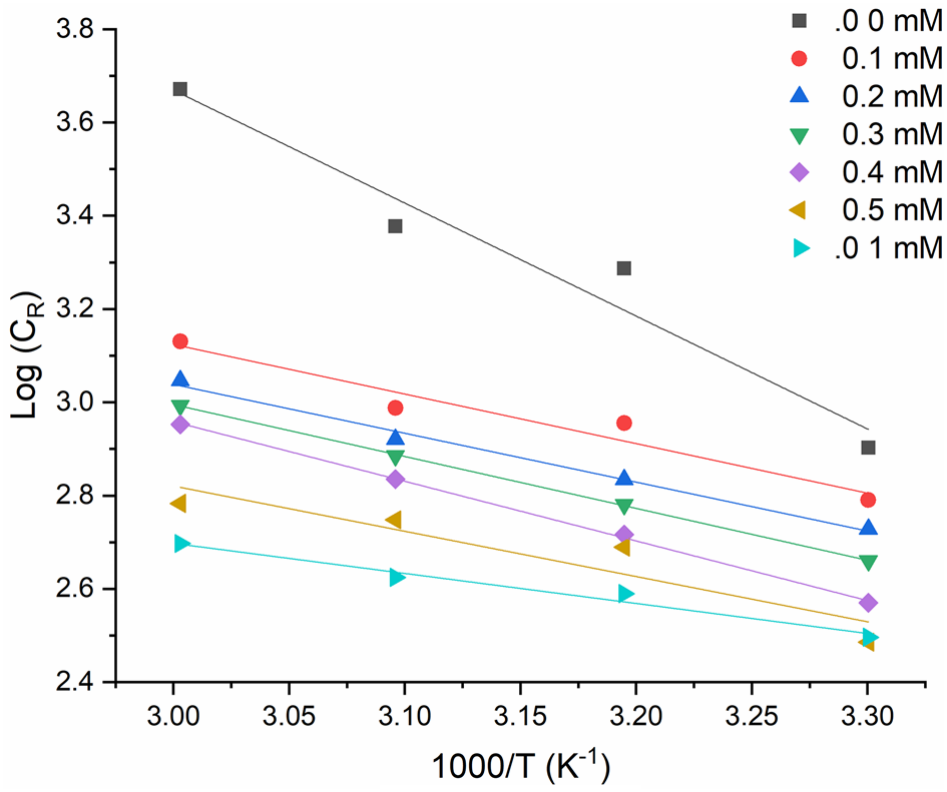
Arrhenius plots for metallic substrate corrosion in 1 M HCl with/without MTIO at various temperatures.

The Arrhenius plot of the logarithm corrosion rate versus ${T}^{-1}$ for the metallic substrate in a corrosive environment with/without various inhibitor concentrations at different temperatures for 5 h is presented in Fig. 5. ${E}_{a}$ value was evaluated according to the slope which is equal to (${E}_{a}$/2.303R) and presented in Table 1. The activation energy (${E}_{a}$) values obtained from the Arrhenius equation for different inhibitor concentrations at 303 K can provide insights into the mechanism of the inhibition process. In general, a higher Ea value indicates a higher energy barrier for the corrosion reaction, which means that the inhibitor is more effective in hindering the reaction. In this study, the Ea values for the inhibited system were found to be higher than those for the uninhibited system at all inhibitor concentrations. This suggests that the MTIO inhibitor provides a higher energy barrier for the corrosion reaction, leading to a lower corrosion rate compared to the uninhibited system. Furthermore, the ${E}_{a}$ values for the inhibited system increased with increasing inhibitor concentration up to 0.5 mM, indicating that the inhibitor's protective effect is enhanced as the concentration increases. However, beyond 0.5 mM, the Ea values decreased, which may be attributed to the saturation of the inhibitor molecules on the metal surface, leading to a decrease in the inhibitory effect. Overall, the results from the Ea values obtained at 303 K are consistent with the weight loss measurements, which showed that the inhibitor's ability to prevent corrosion increased with increasing concentration up to 0.5 mM and decreased at higher concentrations.

**Table 1:** Metallic substrate activation parameters in corrosive environment without and with the addition of MTIO.

Parameter	0.0 mM	0.1 mM	0.2 mM	0.3 mM	0.4 mM	0.5 mM	1 mM
Intercept	10.93	6.31	6.18	6.33	6.79	5.73	4.62
Slope	− 2.42	− 1.06	− 1.04	− 1.11	− 1.27	− 0.97	− 0.64
Ea (kJ.mol^-1^)	12.25	18.57	19.91	20.29	21.25	24.31	26.33

The kinetic-dynamic model was utilized to evaluate the interactions of the MTIO particles with the metallic substrate. Activation enthalpy (∆H) and activation entropy (∆S) were calculated for the activation complex formation in the transition state relation using the experimental values of the ${C}_{R}$ from the weight loss results. The transition state is expressed in Eq. (15):

15 $${C}_{R}=\frac{RT}{Nh}exp\left(\frac{{\Delta S}^{*}}{R}\right)exp\left(\frac{{-\Delta H}^{*}}{RT}\right)$$

where ${C}_{R}$ is the corrosion rate, h refers to Plank constant, and N is the number of Avogadro.

**Figure 6:**
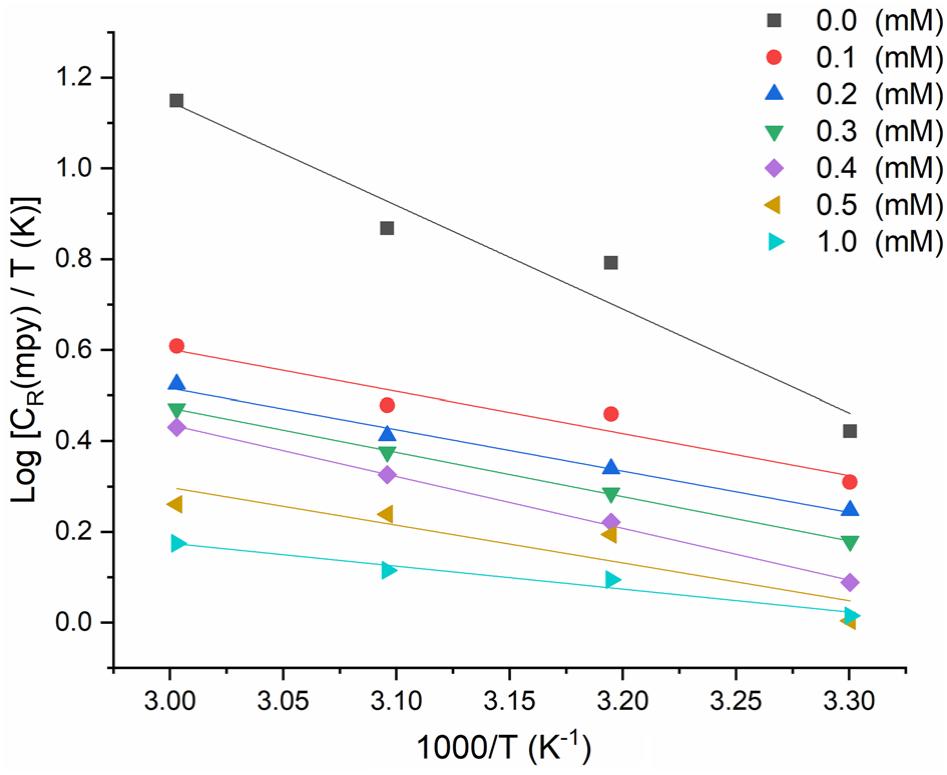
Kinetic-thermodynamic plots for metallic substrate corrosion in HCl without and with MTIO at different temperatures.

Figure 6 shows the plots of log(${C}_{R}$/T) versus 1/T, and the parameters ∆H* and ∆S* were evaluated from the slope (${\Delta H}^{*}$/2.303R) and intercept [$log(R/Nh)+({\Delta S}^{*}/2.303R)$]. Table 2 provides a list of the kinetic-thermodynamic variables^45–47^. The ∆S values vary negatively with and without the inhibitor, suggesting that the association rather than dissociation, which indicates greater ordering, produces the activated complex in the rate-determining phase. These results are consistent with the previous findings from weight loss measurements, where the inhibition efficiency increased with increasing temperature and concentration, indicating that the inhibitor molecules adsorb onto the metallic substrate's surface to form a protective layer. Additionally, the positive ∆H* values suggest that the adsorption of the inhibitor molecules onto the metallic substrate's surface is an endothermic process, indicating that the adsorption occurs via physisorption rather than chemisorption.

**Table 2:** Entropy and enthalpy activation values change at different activation concentrations.

Parameter	$0.0\,mM$	$0.1\,mM$	$0.2\,mM$	$0.3\,mM$	$0.4\,mM$	$0.5\,mM$	$1\,mM$
${\Delta \mathrm{H}}^{*}{ (\mathrm{kJ}.\mathrm{mol}}^{-1})$	51.45	48.39	41.37	38.51	33.49	28.47	22.73
${\Delta \mathrm{S}}^{*}{ (\mathrm{kJ}.\mathrm{mol}}^{-1}{.\mathrm{K}}^{-1})$	− 5.38	− 29.44	− 58.48	77.25	− 101.9	− 117.38	− 138.97

#### Adsorption isotherm

**Figure 7:**
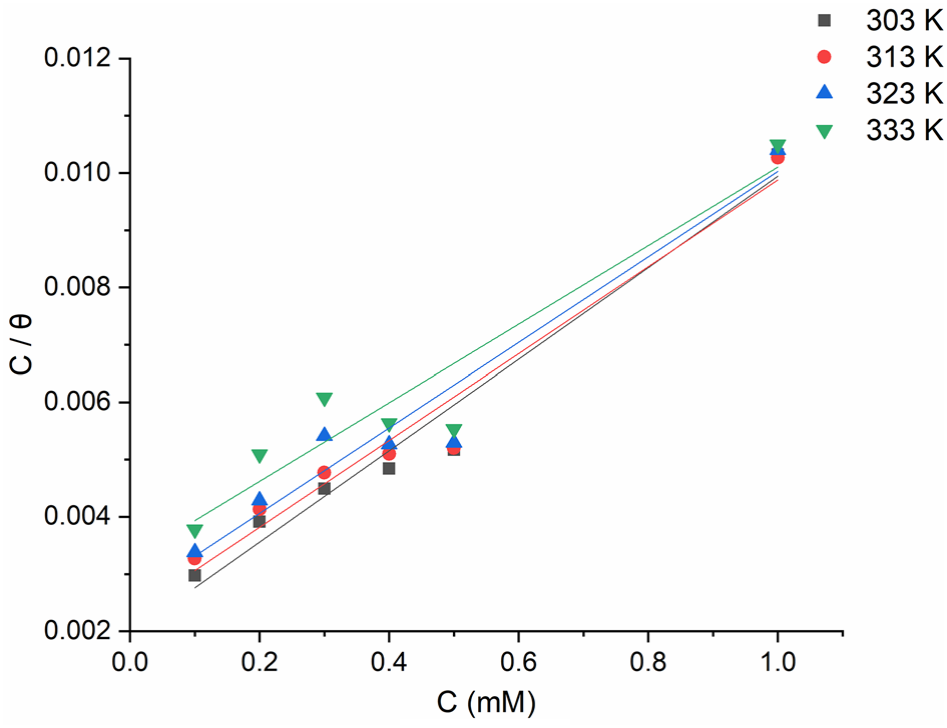
Langmuir adsorption isotherm plots for mild steel immersed in 1 M HCl for 5 h in the presence of various concentrations of MTIO at different temperatures.

MTIO molecules adsorption onto the metallic surface protect it from corrosion, therefore, understanding the inhibition process of MTIO makes the research of the adsorption isotherm extremely important. The percentage surface covering was determined and three adsorption isotherms, namely Frumkin, Temkin, and Langmuir were evaluated to identify the adsorption mechanism. After analyzing the experimental data, it was found that the Langmuir isotherm was the most appropriate model to fit the adsorption data (Fig. 7). The Langmuir isotherm exhibited a linear relationship with a regression coefficient (R2) value close to unity. The Langmuir adsorption isotherm equation (Eq. 4 in section “Adsorption isotherm”) is commonly used to describe the adsorption behavior of monolayer adsorbents^48^.

The ${K}_{ads}$ value was calculated from the Langmuir isotherm plot shown in Fig. 7, which displays a linear relationship between $log (C/\theta )$ and ${C}_{inh}$. The calculated ${K}_{ads}$ value is presented in Table 3. The ${K}_{ads}$ value indicates how effectively an inhibitor molecule adheres to a metallic substrate, thus the higher the ${K}_{ads}$, the greater the adsorption and more effective inhibition^49^. The tested inhibitor had the greatest ${K}_{ads}$ value, indicating that its adsorption on mild steel surfaces will be the greatest. Based on the connection^50^, the ${K}_{ads}$ values and the standard free energy of adsorption (${\Delta G}_{ads}^{0}$) are related (Eq. 16):

**Table 3:** The thermodynamic variables which determined based on weight loss analysis for mild steel in HCl without and with the addition of tested corrosion inhibitor.

Isotherm	Parameters	303 K	313 K	323 K	333 K
Langmuir	R^2^	0.98424	0.98042	0.97206	0.95261
	${K}_{ads}$	135.72	121.26	91.07	77.58
	${\Delta G}_{ads}^{0}$(kJ mol^-1^)	$-34.48$	$-35.88$	$-38.23$	-$39.92$
Temkin	R^2^	0.774	0.753	0.688	0.497

16 $${\Delta G}_{ads}^{0 }=-RTln(55.5{K}_{ads})$$

Typically, physisorption (physical adsorption) occurs between inhibitor molecules and the mild steel surface when the free energy change of adsorption (${\Delta G}_{ads}^{0}$) is less negative than $-20$ ${kJ mol}^{-1}$. In contrast, chemisorption (chemical adsorption), which involves the formation of a coordination bond between the inhibitor and the metallic surface, typically occurs when ${\Delta G}_{ads}^{0}$ values are more negative than $-40$ ${kJ mol}^{-1}$. This process may involve the unpaired electron transfer of heteroatoms from the inhibitor molecules to the d-orbitals of iron atoms on the surface^35,51,52^. The ${\Delta G}_{ads}^{0}$ in this study ranged from $-34.48$ to $-39.92$ ${kJ mol}^{-1}$, indicating a wide range of adsorption (involving both physisorption and chemisorption). Since they lessen the metal's natural reactivity at the locations where they are connected, chemisorbed molecules are expected to offer more effective protection. However, the G_ads_ value alone makes it impossible to distinguish between chemisorption and physisorption. Furthermore, the physical adsorption of inhibitors on the metal surface occurs before their chemical adsorption^53,54^. The values were positive and increased with an increase in temperature. With the increase in concentration the free energy of activation increases and ascribed to the formation of unstable activated complex in the rate determining transition state.

### Electrochemical measurements

#### EIS

**Figure 8:**
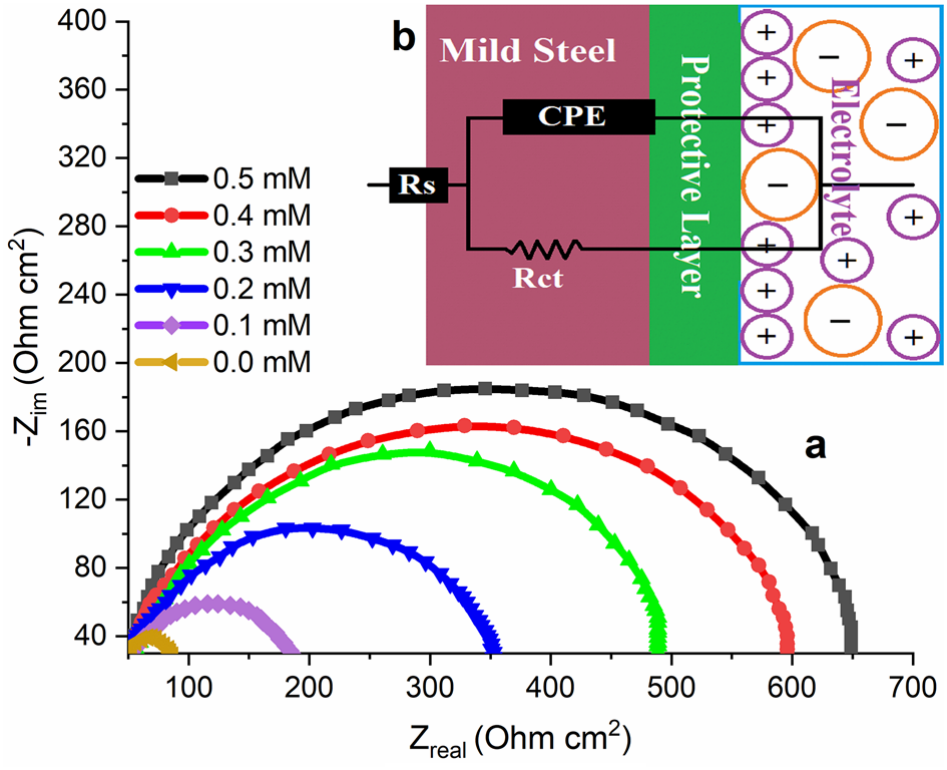
(**a**) Metallic substrate Nyquist plots in uninhibited and inhibited solutions. (**b**) Equivalent circuit applied to EIS data fitting.

EIS provides data on the solution/metal interface capacitive and resistive behaviour without and with different concentrations of the investigated inhibitor^55^. The Nyquist plots of metallic substrate in 1 M HCl in presence and absence of different MTIO concentrations initiated after 30 min exposure period are demonstrated in Fig. 8(a) and are comparable, indicating that MTIO reduces corrosion without modifying its mechanism^55^. Nyquist diagrams of solid metal electrodes due to interfacial impedance frequency dispersal consist of low semicircles with real axis centres. The surface roughness, electrode surface inhomogeneity, electrode breakage, and adsorption of MTIO molecules and impurities are usually attributed to this occurrence^56^ and are represented in Fig. 8b. The circuit consists of the ${R}_{s}$ (resistance of the solution), a CPE (constant phase element) and the Rct (resistance of charge transfer). In this instance, CPE was employed in place of pure double-layer capacitance (${C}_{dl}$) to consider the impact of impurities, the formation of the porous layer, dislocation, the adsorption of inhibitors, and grain boundaries on the mild steel surface^57,58^. The CPE impedance (ZCPE) was computed using Eq. (17)^59^:

17 $${Z}_{CPE}=\left(\frac{1}{{Y}_{0}}\right){\left[{(j\omega )}_{n}\right]}^{-1}$$

where Y_0_ is the constant of the constant phase element, j refers to an imaginary number, ω signifies the angular frequency, and **α** is the phase shift (surface roughness measure).

CPE can indicate impedance ($\boldsymbol{\alpha } = 0$), capacitance ($\boldsymbol{\alpha } = 1$), inductance ($\boldsymbol{\alpha } = 1$), or Warburg resistance ($\boldsymbol{\alpha } = 0.5$) depending on the value of n. Equation (18) was used to determine the ${C}_{dl}$ values without and with the addition of the inhibitor^60^:

18 $${C}_{dl}={Y}_{0}{\left[{\omega }_{max}\right]}^{n-1}$$

where the maximum (${rads}^{-1}$) value of the imaginary component of resistance is reached at frequency ω_max_.

Table 4 provides a list of the measured variables including ${R}_{s}$, ${R}_{ct}$, α, ${C}_{dl}$, IE%, and surface coverage (θ). There was a considerable increase in the ${R}_{ct}$ value with an increase in the MTIO concentration, indicating that the inhibitor slowed the rate of the charge transfer reaction by adsorbing onto the metallic substrate, thereby slowing the corrosion rate^61^. Also, the ${C}_{dl}$ values were lower in the inhibiting solution than in the uninhibited solution possibly because of reduced local dielectric constant and/or an increased electrical double-layer thickness, confirming that the adsorption process prevents metallic substrate corrosion^62^. The n value in the inhibited solution is higher than that in the uninhibited one, and they are almost constant and close to unity, indicating that the surface is smoother in the solution containing the inhibitor, which is caused by the metallic substrate surface being protected by the protective barrier adsorbed onto the surface^63^.

**Table 4:** EIS Parameters values and IE for metallic substrate in uninhibited and inhibited solutions.

$C\,(mM)$	${R}_{s}(\Omega\,{cm}^{2})$	${R}_{ct}(\Omega\,{cm}^{2})$	CPE_dl_		${C}_{dl}(\mu F.{cm}^{-2})$	$IE\%$
			α	Y_o_ (µS.s^α^ cm^-2^)		
0	0.234 ± 0.04	9.47	0.910	0.0011	4.184	–
0.1	0.351 ± 0.02	77.49	0.861	0.0045	0.634	70.4
0.2	0.368 ± 0.01	132.48	0.778	0.0055	0.538	77.2
0.3	0.413 ± 0.04	185.66	0.883	0.0006	0.515	84.9
0.4	0.523 ± 0.03	258.34	0.891	0.0039	0.407	92.4
0.5	0.603 ± 0.07	220.17	0.887	0.0057	0.310	96.4

#### Potentiodynamic polarization curve

**Figure 9:**
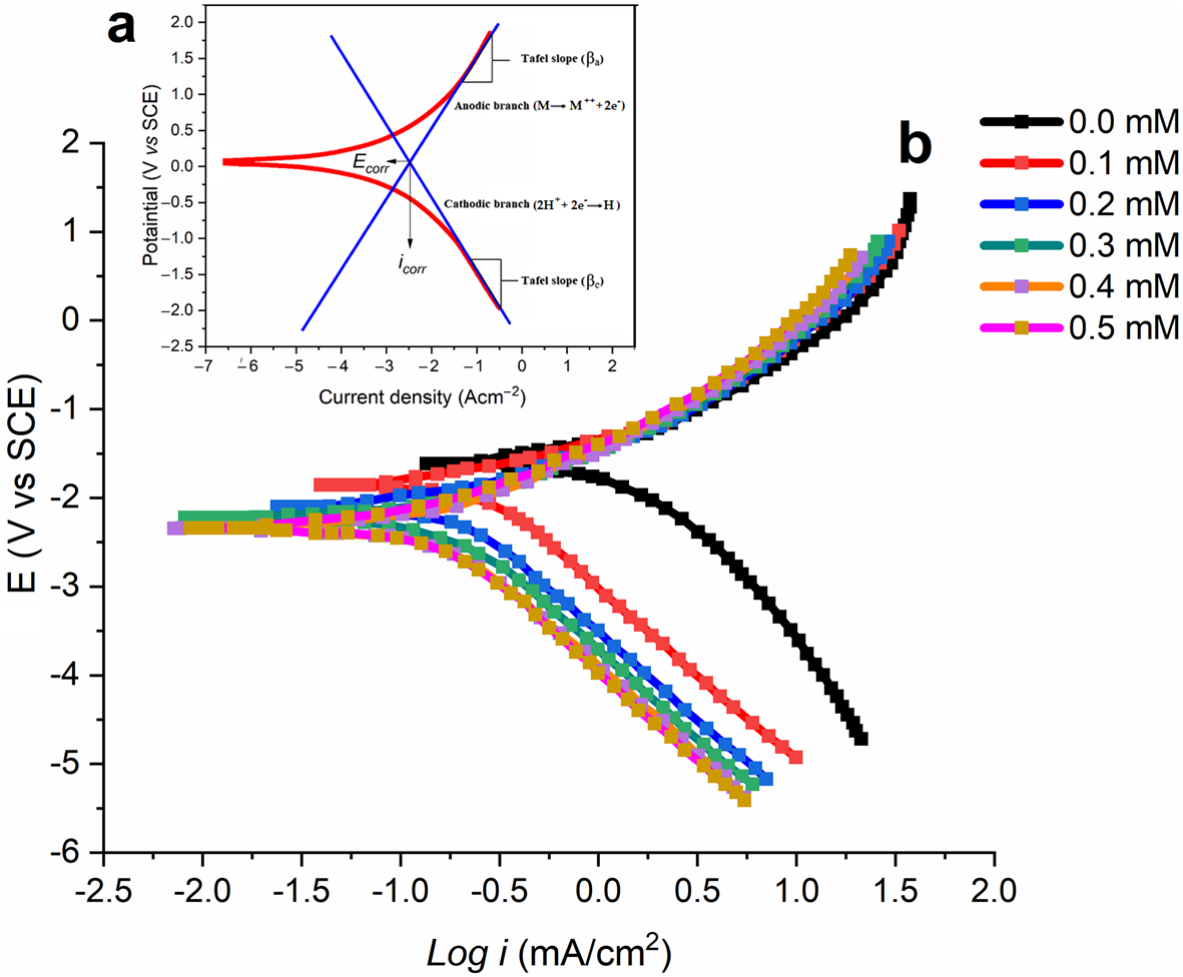
(**a**) The extrapolation of the Tafel slopes. (**b**) Metallic substrate polarization curves in uninhibited and inhibited solutions.

Tafel projection is important for corrosion and is employed in polarizing approaches such as potentiodynamic measurements, cyclic polarisation, and linear polarisation impedance. Observations of polarisation impedance can be utilised to evaluate the corrosion dynamics of thin films (and possibly coating layers) over time. The main parameter utilized to evaluate the kinetic efficacy of protective coatings is the ${i}_{corr}$, with higher current densities leading to lower electrochemical behavior. Furthermore, the narrow passivation potential range coupled with the low pitting potential and rapid corrosion rate indicates coating defects and pores that allow diffusion of the electrolyte, facilitating the loss of the protective coating^64^. The Tafel extrapolation method is a popular polarisation technique for estimating ${C}_{R}$. Compared to standard weight loss measurements, this technique (referring to the specific technique being discussed) is faster and provides more accurate results. However, it is important to note that the corrosion rate (${C}_{R}$) calculated using Tafel extrapolation of polarization curves may differ from those calculated using weight loss, as the two methods measure different aspects of corrosion The Tafel formula shows a mixed-potential theory that leverages the kinetics and thermodynamics of all events taking place at the electrode surface to forecast ${C}_{R}$ and potentials since it has the ability to refer to a range of corrosion-related reactions. Corrosive situations are often excluded from the reversible potential of all processes. Tafel kinetics therefore provides a correct description of corrosion kinetics in situations where mass transfer limitations are not considered. When a metal electrode is immersed in a corrosive water environment, anodic and catalytic reactions spontaneously occur on the electrode surface, leading to corrosion of the electrode. In this case, the subsequent electrode potentials and the reversal or equilibrium potentials of each reaction occurring at the surface cannot be compared. The ${i}_{corr}$ is determined by deriving the linear component of the curve to ${\mathrm{E}}_{\mathrm{corr}}$, as demonstrates in Fig. 9. Assuming uniform corrosion, Faraday's law can be used to convert ${i}_{corr}$ to penetration rate or weight loss, allowing not only to measure significantly lower corrosion rates but also to continuously monitor the system under investigation. Figure 9 shows the Nyquist and Bode plots of mild steel in 1 M HCl in the presence and absence of MTIO at different concentrations. Figure 9 shows the procedure and Eq. (19) shows how the inhibitory effect was evaluated.

19 $$IE\left(\%\right)=\frac{{i}_{corr}-{i}_{corr(inh)}}{{i}_{corr}}\times 100$$

where ${i}_{corr}$ and ${i}_{corr(inh)}$ are the currents in uninhibited and inhibited solutions.

Figure 9 at 303 K displays the polarisation curves for the metallic substrate specimen in uninhibited and inhibited solutions with various inhibtor concentrations. Table 5 displays the information for the corrosion potential (${E}_{corr}$), corrosion current density (${i}_{corr}$), and IE along with the anodic (${\beta }_{a}$) and cathodic (${\beta }_{c}$) Tafel slopes. The Gamry-E chem Analyzer software presents the Tafel fit method, which uses a nonlinear chi-square minimizing to match the information to the Stern-Geary formula.

**Table 5:** Metallic substrate Tafel parameters in uninhibited and inhibited solutions.

*C mM*	${E}_{corr}$ ($mV$)	${\beta }_{a}$ ($mV/dec$)	${-\beta }_{c}$ ($mV/dec$)	${i}_{corr}$ ($\mu A{cm}^{-2}$)	$IE$ $(\%)$
0.0	–472	80.4	130.4	670	0
0.1	–515	73.5	121.5	312.5	62.7
0.2	–522	70.6	119.7	195.7	71.2
0.3	–545	68.4	117.9	148.1	78.9
0.4	–532	64.5	111.3	119.4	98.8
0.5	–548	54.1	109.5	113.5	96.1

The dispersal of ${\mathrm{E}}_{\mathrm{corr}}$ by MTIO occurs primarily when the ${\mathrm{E}}_{\mathrm{corr}}$ shift reaches 85 mV. This suggests that the corrosion inhibitor could be classified as having both cathodic and anodic properties. As a result, the particles could be considered as having a mixed-type behavior. In the presence of MTIO in the acidic media, the cathodic hydrogen generation is postponed, and the anodic degrading of metallic substrate is decelerated. According to Table 5, where the addition of MTIO resulted in a decline in current density readings, the ${C}_{R}$ decreased as the MTIO concentration increased, strengthening the inhibitory efficacy. Both processes were under the influence of the MTIO, as shown by the ${\beta }_{a}$, and ${\beta }_{c}$, which were rarely altered while the MTIO was present. Particles had no effect on the development of hydrogen or the dissolution of metallic substrate^65^. In an acid medium, MTIO successfully stopped or retard the corrosion of metallic substrate^66–68^. Following are the metallic substrate and cathode reactions in HCl, and relation (20) illustrates the cathodic reaction's process.

20 $${2\mathrm{H}}^{+}+{2\mathrm{e}}^{-}={\mathrm{H}}_{2}\uparrow$$

The anodic reaction mechanism in uninhibited solution (Eqs. 21–24):

21 $$\mathrm{Fe}+{\mathrm{Cl}}^{-}\leftrightarrow ({\mathrm{FeCl}}^{-}{)}_{ads}$$

22 $$({\mathrm{FeCl}}^{-}{)}_{ads}\leftrightarrow (\mathrm{FeCl}{)}_{ads}+{e}^{-}$$

23 $${2\mathrm{H}}^{+}+{2\mathrm{e}}^{-}={\mathrm{H}}_{2}\uparrow$$

24 $${\mathrm{FeCl}}^{+}+{\mathrm{e}}^{-}\leftrightarrow {\mathrm{Fe}}^{++}+{\mathrm{Cl}}^{-}$$

The anodic reaction mechanism in inhibited solution (Eqs. 25–30):

25 $$\mathrm{Fe}.{\mathrm{H}}_{2}{\mathrm{O}}_{ads}+inh\leftrightarrow {\mathrm{FeOH}}_{ads}^{-}+{\mathrm{H}}^{+}+inh$$

26 $$\mathrm{Fe}.{\mathrm{H}}_{2}{O}_{ads}+inh\leftrightarrow {\mathrm{Fe}.inh}_{ads}+{\mathrm{H}}_{2}\mathrm{O}$$

27 $${\mathrm{FeOH}}_{ads}^{-}\to {\mathrm{FeOH}}_{ads}+{\mathrm{e}}^{-}$$

28 $${\mathrm{Fe}.inh}_{ads}\leftrightarrow {\mathrm{Fe}.inh}_{ads}^{+}+{\mathrm{e}}^{-}$$

29 $${{\mathrm{FeOH}}_{ads}+\mathrm{Fe}.inh}_{ads}^{+}\leftrightarrow {\mathrm{FeOH}}^{+}+{\mathrm{Fe}.inh}_{ads}$$

30 $${\mathrm{FeOH}}^{+}+{\mathrm{H}}^{+}\leftrightarrow {\mathrm{Fe}}^{+}+{\mathrm{H}}_{2}\mathrm{O}$$

The presence of MTIO produces a film of $\mathrm{Fe}.{inh}_{ads}$ that efficiently prevents the deposition of anodic iron ions and reduces the charges at the interface between metallic substrate and acidic medium, which severely limits the formation of the cathodic hydrogen reaction. The polarisation data unequivocally demonstrate that the MTIO mostly has a cathodic effect.

### Surface morphology

**Figure 10:**
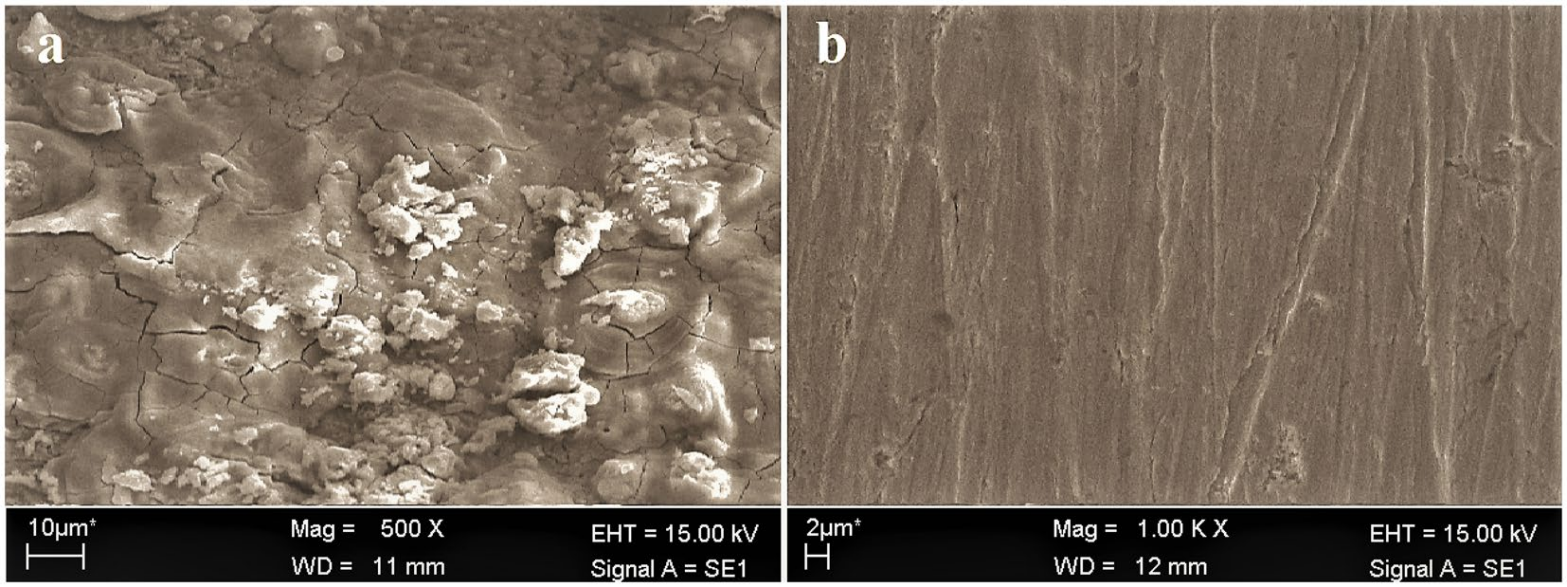
SEM photographs in 1 M HCl without (**a**) and with (**b**) MTIO.

SEM pictures of the metallic substrate surface strip during 5 h of exposure in uninhibited and inhibited hydrochloric acid solution with MTIO are shown in Figs. 10a and b. As seen in Fig. 10a, the surface was sagging and crowned, which indicated severe corrosion of the metallic substrate surface. Figure 10b depicts the surface characteristics with substantially less corrosion than Fig. 10 with the addition of MTIO (a).

### Theoretical calculations

#### DFT

**Figure 11:**
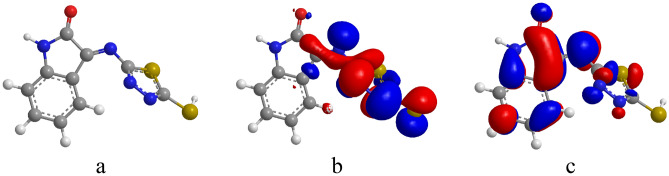
The (**a**) 3rd-structue, (**b**) HOMO and (**c**) LUMO of MTIO molecule.

The LUMO indicates the ability to accept electrons and is electron acceptor, whereas the HOMO indicates the ability to provide electrons and possesses electrons. As EHOMO rises and ELUMO drops, the inhibitor particles' capacity to adsorb on the metallic substrate surface increases. Figure 11 shows the sight of the MTIO molecule's 3rd structure, while Table 6 lists the EHOMO and ELUMO.

**Table 6:** Quantum parameters for the MTIO molecules in gas phase.

${E}_{\mathrm{HOMO}}$	${E}_{\mathrm{LUMO}}$	$\Delta E$	$I$	$A$	$\chi$	$\eta$	$\sigma$	$\Delta N$	µ
− 8.106	− 4.237	− 3.869	8.106	4.237	6.1715	1.9345	0.5169	0.428	− 2.3434

Computed results are in reasonable agreement in that they imply that the chemical structure of MTIO is quite-organized and extremely quite-adsorbed on the metallic substrate, accomplishing a wider coverage surface area. The thiadiazole side ring extends out of the plane in the energy-optimized molecular geometry for MTIO, whereas the isatin ring is plane (Fig. 11a), making the flat configuration of the thiadiazole and isatin rings ideal for adsorption on the metallic substrate. According to Fig. 11b, the electron densities in HOMO are dispersed over the thiadiazole ring and a small proportion of the isatin ring. Additionally, Fig. 11c demonstrates that the electron densities in LUMO are dispersed throughout the molecule, which facilitates electron transfers from MTIO particles to vacant 3d metal orbitals and from entire 4 s metal orbitals to LUMO^69^. The transfer of electrons from inhibitor molecules slows the pace at which anodic metal dissolves while increasing the electronic structure on the anodic locations of the metallic substrate. The cathodic sites where hydrogen ions are shocked, though, suffer from a lack of electronic structure due to retro-electron donation.

The MTIO molecules' mixed-type inhibiting properties can therefore be described by a straightforward two different electrical transmission. A passive metal ion-inhibitor coating forms on the surface of metallic substrate specimen as a result of MTIO molecules' binding of Fe-atoms to form a metallic complexes, which prevents further corrosion. The heterocyclic ring may have a substantial impact on the whole adsorption process even if it is not intimately implicated in charge transfer during adsorption. Although the energy gap (∆E) is normally expanding, the EHOMO is significant and the ELUMO is remarkably low. As shown by the electronic donation fraction (∆N), the impact of donating electrons from the MTIO molecules to the Fe is comparatively quite pervasive than that of the retroactive donation from the steel to the inhibitor. According to Elnga and colleagues., whenever ∆N is positive, electrons donating to the iron^70^, facilitated by MTIO's reduced electronegativity (χ), boosts the inhibitory performance of the MTIO. If other compound global softness (σ) is greater, MTIO can interact with them in increasingly intricate ways. The dipole moment is a significant additional factor that contributes to enhanced electrostatic relationship between two interrelated components. The stronger the electrostatic contact between MTIO and the various metal ions substrate, the larger the MTIO dipole moment.

#### Mulliken charges

It is regular procedure to quantify atomic charges within compound and find inhibitor active site using Mulliken charges. Additionally, a heteroatom's potential for donation adsorption onto a metal substrate increases with the negative charge of the atoms. Because of their significant atomic charges (S(17) = −0.862, N(1) = −0.123, N(14) = −0.377, O(10) = −0.467), the nitrogen, and oxygen, atoms are thought to be responsible for adsorbing metal. Mulliken charges of the MTIO are displayed in Table 7.

**Table 7:** Shows the Mulliken charges of MTIO.


Atoms	Charges	Atoms	Charges	Atoms	Charges	Atoms	Charges
[N(1)]	− 0.123	[C(6)]	0.379	[N(11)]	0.151	[C(16)]	0.061
[C(2)]	0.019	[C(7)]	− 0.269	[S(12)]	1.693	[S(17)]	− 0.862
[C(3)]	0.147	[C(8)]	− 0.172	[C(13)]	− 0.524	[H(18)]	0.291
[C(4)]	− 0.171	[C(9)]	0.109	[N(14)]	− 0.377	[H(19)]	0.029
[C(5)]	− 0.138	[O(10)]	− 0.467	[N(15)]	0.046	[H(20)]	0.025

#### Mechanism of inhibition

**Figure 12:**
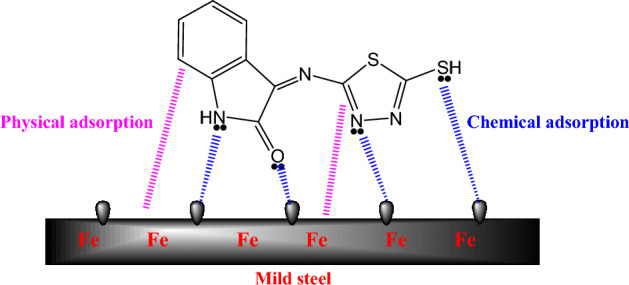
Suggested inhibition mechanism of metallic substrate in an acid solution.

The adsorption of inhibitor molecules onto the metallic surface is influenced by several factors, including their chemical structure, atomic charges, behavior in the acid medium, and interaction with the surface properties of the metallic substrate. EIS, PDP and weight loss techniques revealed that the MTIO particles considerably retard/inhibit the metallic substrate corrosion. The adsorption isotherm explorations^71–73^ also confirmed the assumptions of the Langmuir adsorption model for how the MTIO particles adsorb to the metallic substrate. Chemical adsorption onto the metallic substrate is facilitated by electron pairs of heteroatoms in the MTIO. Figure 12 depicts the proposed mechanism for metallic substrate corrosion protection in an acidic medium.

## The advantages of this work

This work aimed to investigate the inhibitory effect of a specific compound, MTIO, on the corrosion of mild steel in hydrochloric acid solution. The study explored the impact of temperature and concentration of the inhibitor on the corrosion behavior using various methods such as weight loss, electrochemical impedance spectroscopy, and potentiodynamic polarization techniques. The results indicated that MTIO showed promising inhibition properties, significantly reducing the corrosion rate of mild steel. Therefore, one of the main problems that this work solved is the development of an effective inhibitor for the corrosion of mild steel in hydrochloric acid solution^74,75^.

### The present work is important for several reasons

First, it investigates the inhibitive properties of MTIO on mild steel corrosion in acidic solutions, which is a relevant topic in the field of corrosion science and engineering^76,77^. Second, it applies various analytical techniques, such as electrochemical impedance spectroscopy, potentiodynamic polarization, and weight loss measurements, to comprehensively evaluate the inhibitive efficacy of MTIO. Third, the study provides insights into the kinetic and thermodynamic aspects of the inhibition process, which can contribute to the development of new and effective corrosion inhibitors. Finally, the results of this study could potentially have practical implications for the selection and application of corrosion inhibitors in various industries, such as oil and gas, construction, and transportation.

### The is importance of this work in the real industries

The importance of this work in real industries lies in the development of new, effective, and environmentally friendly inhibitors for the protection of mild steel in acidic media. Mild steel is widely used in various industrial applications, and its corrosion can result in significant economic losses. By developing effective inhibitors, the industries can prevent corrosion and extend the service life of mild steel, resulting in cost savings and improved operational efficiency. Additionally, the use of environmentally friendly inhibitors aligns with the growing demand for sustainable and eco-friendly practices in the industry. Overall, this work can contribute to the development of more efficient and sustainable corrosion protection strategies in the real industries.

### Comparsion with the old works

The novel contributions and advancements made in the new work are:

- The use of a new inhibitor, MTIO, to protect mild steel from corrosion in acidic media.
- The investigation of the inhibitory properties of MTIO using a combination of weight loss, electrochemical, and surface analysis techniques.
- The determination of the kinetic-thermodynamic parameters of the corrosion process and inhibition mechanism using Tafel polarization curves.
- The observation of MTIO particles adhering to the surface of the specimen through the drop in C_dl_ and rise in R_ct_ in electrochemical impedance spectroscopy (EIS) tests.
- The comparison of the inhibitory properties of MTIO with other known inhibitors, such as benzimidazole, imidazole, and thiourea.
- The evaluation of the effect of temperature and concentration on the inhibition efficiency of MTIO.
- The investigation of the surface morphology and composition of the mild steel specimens before and after exposure to corrosive media using scanning electron microscopy (SEM) and energy-dispersive X-ray spectroscopy (EDX).

These contributions represent advancements in the field of corrosion inhibition and provide new insights into the use of MTIO as an effective inhibitor for the protection of mild steel in acidic media.

## Conclusions

MTIO exhibits significant barrier properties for metallic substrate specimens in corrosive media due to the highly efficient and effective adsorption centers present, such as O, N, S, and pi-bonds. These centers impede the active locations on the surface of the specimen. The following are the main conclusions:

- In the corrosive media, MTIO demonstrated excellent metallic substrate anticorrosion efficacy with an inhibitor efficiency of 96.9% at 303 K, as determined by the weight loss method.
- MTIO particles chemically adsorb and weakly bind to metal substrates, with the inhibiting performance diminishing as the temperature increases. The inhibiting performance increases with rising MTIO concentration and reduces with increasing temperature. At 303 K in a 1 M HCl solution, the greatest inhibitory performance was 96.9%.
- The ${\Delta G}_{ads}^{0}$ model predicts spontaneous chemical and physical adsorptions. MTIO reduces metallic substrate corrosion by generating a protective film of MTIO particles at the steel-electrolyte interface.
- The decrease in the double-layer capacitance (${C}_{dl}$) and the increase in charge-transfer resistance (${R}_{ct}$) indicate that the MTIO particles adhere to the surface of the specimen, forming a protective layer that hinders the corrosion process. This behavior is consistent with the results obtained from other corrosion measurement techniques, such as weight loss and polarization curves, which also showed that the MTIO inhibitor was able to effectively protect the metallic substrate against corrosion. Therefore, the conclusion drawn from this point is that the MTIO particles adhere to the surface of the specimen and form an effective protective layer that contributes to the excellent anticorrosion efficacy of the MTIO inhibitor.
- Additionally, SEM pictures show that the MTIO prevents the metal surface from corrosive attack.
- The quantum chemical computations show that MTIO uses oxygen, sulphur, and nitrogen to adsorb onto the tested specimen surface. The outcomes of the simulations analysis were in agreement with the laboratory observations and gave understanding into the electronic structure of MTIO particles and their adsorption behaviour on the metal substrate.

## Data Availability

All data generated or analysed during this study are included in this published article.
